# Cognitive Impairment in People with Epilepsy

**DOI:** 10.3390/jcm11010267

**Published:** 2022-01-05

**Authors:** Ajda Novak, Karmen Vizjak, Martin Rakusa

**Affiliations:** Department of Neurologic Diseases, University Medical Centre Maribor, 2000 Maribor, Slovenia; ajda.novak95@gmail.com (A.N.); karmen.vizjak@gmail.com (K.V.)

**Keywords:** epilepsy, cognitive impairment, seizures, anti-epileptic drugs, memory deficits, EEG

## Abstract

People with epilepsy frequently have cognitive impairment. The majority of cognitive problems is influenced by a variety of interlinked factors, including the early onset of epilepsy and the frequency, intensity and duration of seizures, along with the anti-epileptic drug treatment. With a systematic review, we investigate significant factors about the cognitive impairment in epilepsy. Most cognitive problems in adult people with epilepsy include memory, attention and executive function deficits. However, which cognitive area is mainly affected highly depends on the location of epileptic activity. Moreover, modifications in signalling pathways and neuronal networks have an essential role in both the pathophysiology of epilepsy and in the mechanism responsible for cognitive impairment. Additionally, studies have shown that the use of polytherapy in the treatment of epilepsy with anti-epileptic drugs (AEDs) heightens the risk for cognitive impairment. It can be challenging to distinguish the contribution of each factor, because they are often closely intertwined.

## 1. Introduction

The International League Against Epilepsy (ILAE) defines epilepsy as a disease of the brain characterized by any of the following conditions: at least two unprovoked (or reflex) seizures occurring >24 h apart; one unprovoked (or reflex) seizure and a probability of further seizures similar to the general recurrence risk (at least 60 %) after two unprovoked seizures, occurring over the next 10 years; diagnosis of an epilepsy syndrome [1,2]. In Fiest et al.’s meta-analysis [3], the overall lifetime prevalence of epilepsy worldwide is 7.6 per 1000 members of the population. The incidence and prevalence are slightly higher in men than in women.

Epileptic seizures are the result of the abnormal functioning of voltage-gated and transmitter-gated ion channels, which can make neurons electrically hyperactive. In general, they can be classified as seizures with focal, generalized, or unknown onset [4]. There are several types of epileptic seizures, which can present as either minor or dramatic, short or long, frequent or rare. Clinically, they can range from a major generalized tonic–clonic seizure to a mild myoclonic flickering of the eyelids or a focal numbness of the thumb and mouth [5]. The symptoms depend on the location, spread and the intensity of the electrical changes. Usually, after the end of the seizure and after the return of consciousness, a postictal period or a period of confusion occurs. This period can last from a few minutes to several hours. The common symptoms are fatigue, headache, speech problems, abnormal behaviour and impaired concentration and memory, which disappear over time [6]. 

People with epilepsy (PWE) are also exposed to many other health problems that occur often. For many people, co-morbidity is more burdensome than the seizures themselves. Epileptic seizures can cause both morphological and functional changes in the brain, manifesting as cognitive and neuropsychological disorders. Frequent seizures, especially status epilepticus, repeatedly cause oxidative stress; neuronal loss, mainly in hippocampus or entorhinal cortex, which is another area closely associated to cognitive processing; neurogenesis; changes in growth factors such as BDNF; and inflammation in the brain [7,8]. If the epileptic seizures are not properly treated and controlled, they can lead to permanent cognitive dysfunction [9]. The difficulty with cognition is one of the most common problems in PWE; however, it is often overlooked. Some studies suggest that between 60% and 70% of people with chronic epilepsy have cognitive impairment [10].

The cognitive skills in PWE may be fully affected or partially affected in individual areas, e.g., mild aprosexia (sustained attention deficit disorder), memory impairment, impaired executive function, slowed psychomotor speed, impaired naming ability and impaired visual–spatial abilities [11,12]. Most cognitive dysfunctions that occur in PWE are related to the underlying aetiology of epilepsy. Acquired causes (e.g., injury, hypoxia, or ischemia) or congenital causes (e.g., tuberous sclerosis, fragile X syndrome, Rett syndrome and Dravet’s syndrome) can also lead to cognitive decline. In addition, cognitive decline may occur due to frequent interictal epileptic activity and recurrent or prolonged seizures. It may also be a consequence of the treatment of epilepsy with AEDs. The cause of cognitive decline is mostly a combination of several different factors [8,13]. 

Temporal lobe epilepsy is the most common cause of focal onset impaired awareness seizures. The main functions of the temporal lobe include speech, learning, memory and affective behaviour. The damage to the structures within the lobe would disrupt the mentioned functions. Memory impairment, anxiety and depression are common in people with temporal lobe epilepsy [14]. In this type of epilepsy, the hippocampus is often severely affected and hippocampal sclerosis is the most common epileptogenic lesion [14]. The hippocampus is thought to be crucial for episodic memory, while other parts of the temporal lobe are more important for semantic memory [15]. More frequent and longer seizures are commonly associated with severe hippocampal atrophy, leading to significant cognitive impairment, especially in the area of memory [16]. 

The second most common form of epilepsy in adults is frontal lobe epilepsy. The frontal lobe primarily supports the functioning of higher-level cognitive processes, which include executive functions and working memory. It also has an effect on motor functions, emotional control and inhibition [17]. Structural and functional impairments, such as focal onset seizures in the frontal lobe, can lead to cognitive and behavioural disorders. In adults with frontal lobe epilepsy, cognitive deficits and behavioural disorders are manifested by impaired attention and difficulty in more complex behaviours, including executive functions (planning, anticipation, organization, initiation, working memory and task performance). 

In this review article, we want to present the latest findings on the impact of epilepsy on cognitive functions. Possible neurophysiological mechanisms for the development of cognitive impairment in epileptic seizures, the impact of epileptiform activity on cognitive impairment, the impact of cognitive impairment on psychosocial factors, the impact of antiepileptic drugs (AED) and the impact of epilepsy on cognitive tests in PWE are introduced. 

## 2. Materials and Methods

### 2.1. Search Strategy and Selection Criteria

**Figure 1:**
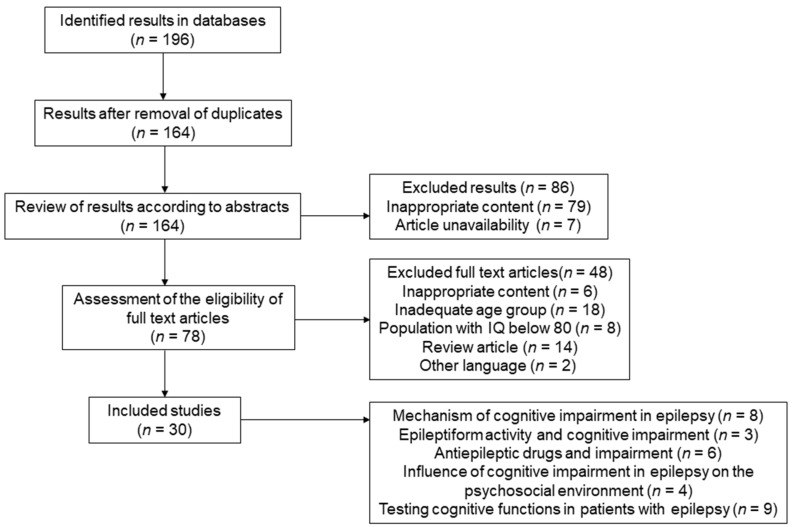
PRISMA literature review flow diagram.

The literature search was conducted according to the Preferred Reporting Items for Systematic Reviews and Meta-Analyses (PRISMA) guidelines (Figure 1) [18] in the PubMed and Google Scholar databases in contents concerning epilepsy and cognitive impairment. We searched for the following keywords and Boolean operators: (“epilepsy” OR “seizures”) AND (“cognition” OR “cognitive dysfunction” OR “cognitive impairment”); (“epilepsy” OR “seizures”) AND (“cognition” OR “cognitive dysfunction” OR “cognitive impairment”) AND (“mechanisms of cognitive impairment” OR “mechanisms of cognitive changes”); (“epilepsy” OR “seizures”) AND (“cognition” OR “cognitive dysfunction” OR “cognitive impairment”) AND (“antiepileptic drug” AND “adverse effects”) throughout the text. 

We limited the results to all original articles published in English from 2005 to 2020, thus excluding papers reviewed in two previous articles on cognitive impairment in PWE [11,19]. All studies that focused on children and adolescents, those that included traumatic head injuries and neurodegenerative disorders as a cause of epilepsy, tumour-associated epilepsy or individuals with developmental disorders were excluded. 

### 2.2. Selection Process

By reviewing the titles, we selected articles that met our requirements in terms of content. Selected articles were entered into the reference management program (Mendeley Ltd., London, UK) and duplicates were removed.

After removing duplicate articles, we reviewed the abstracts of 164 articles. We excluded all those that were not substantively related to cognitive impairment in epilepsy, or those in which cognitive impairment was only indirectly mentioned. There were 78 articles left. Following the exclusion criteria, 49 full text articles were excluded after the review. These were studies with the content or the scope of research that was not directly related to epilepsy, studies that focused only on children and adolescents, studies on the population with an average IQ below 80 and review articles. 

Original articles describing the possible mechanisms of cognitive changes in animals or humans with epilepsy, as well as the impact of epileptic activity on the EEG, were included. Furthermore, studies that investigated the effect of AEDs on cognitive impairment and studies that investigated the impact of cognitive changes on psychosocial factors in everyday lives of PWE were included. Moreover, studies that focused on cognitive functions in adults or the elderly with epilepsy were included.

## 3. Results

Based on the selected search strategy and defined criteria, eight studies related to the mechanism of cognitive impairment in epilepsy, three studies on the effect of epileptic activity on cognitive impairment, six studies on the effect of AED on cognitive impairment, four studies on the association between psychosocial factors and cognitive impairment in epilepsy and nine studies directly related to the testing of cognitive functions in PWE were included (Table 1). 

## 4. Discussion

### 4.1. Mechanisms of Cognitive Impairment in Epilepsy

The changes in cognitive functions often depend on the characteristics of the seizure. Each brain function includes several different spatially distributed areas, which are interlinked and form brain networks. The interaction among aetiology, seizures and cognition is complex [20]. Cognitive skills are significantly affected by the anatomical location of the epileptic activity. Cognitive impairment in adults is largely associated with an area of the brain where the main focus of the epileptic seizure is [20,21]. Thus, adults with temporal lobe epilepsy have been found to have the most impaired memory [22], as expected. The hippocampus plays a key role in shaping the memory. In the case of an epileptic seizure, it has the lowest threshold of abnormal excitability in the brain of all brain structures [23]. Surviving pyramidal cells in the hippocampus form into an abnormal hippocampal circuitry, leading to the impaired long-term, short-term and spatial memory [20].

In many cases, cognitive impairment is most severe in people with early onset epilepsy, especially in epilepsy syndromes, e.g., those classified as epileptic encephalopathies [20]. Epileptic encephalopathy refers to a group of severe epilepsies, which can occur at birth and cause very frequent epileptic seizures, as well as cognitive, neurological and behavioural disorders, and may worsen developmental problems in children [24]. Besides frequent and often severe seizures, these patients generally receive higher doses of AEDs and polytherapy. All these factors can worsen cognitive impairment [20]. 

Additionally, the underlying aetiology in epileptic encephalopathies can often be genetic. In four individual studies, a genetic link to the mechanism of cognitive impairment in epilepsy has been explored. Numerous gene mutations have been found to cause ion channel failure or abnormal cortical development, such as sodium voltage-gated channel alpha subunit 1 (SCN1A) [25], gamma-aminobutyric acid type A receptor subunit alpha1 (GABRA1) [26], tuberous sclerosis-1 gene (TSC1) [27] and potassium sodium-activated channel subfamily T member 1 (KCNT1) [28]. The defects cause areas of the brain to become hyper-excited through a variety of mechanisms, such as changes in synaptic functions, changes in neuronal connectivity, impaired metabolism and impaired homeostasis [29]. The long-term hyper-excitation, regardless of the path of the onset, leads to the reduced neural function and cognitive impairment [8,9].

Furthermore, recurrent epileptic seizures can have a serious impact on cognition. In addition to the inabilities that occur during a seizure, the postictal state is usually a period with reduced cognitive functions. Once the symptoms of lethargy and confusion subside, cognitive impairment may persist for several minutes to several days, depending on the type and the severity of the seizure. When seizures occur frequently, a cumulative degradation in the capacity of spatial memory appears [23]. The exact cause of the postictal impairment has not yet been found. Some studies on animals [23,24,30,31,32] showed that chronic seizures cause the impairment of long-term potentiation (LTP) and affect the accuracy and the stability of place cells. The place cells are located in the hippocampus. They become active at a specific location in space and participate in episodic memory. LTP is a reflection of a steady increase in synaptic power and is crucial for many behavioural adjustments, such as learning and memory, the functional development of vision and the somatosensory system [33]. 

Four studies [23,30,31,32] tested the effect of epileptic seizures on cognitive functions and the association with the mechanism via LTP impairment in mice. Zhou et al. [30] and Lin et al. [23] measured the spatial abilities in healthy adult mice in two separate studies. In the first study, the seizures in mice were triggered by flurothyl for eleven consecutive days, while, in the second study, the seizures were triggered twice a day for five consecutive days. They discovered that the spatial abilities in mice deteriorated in proportion to the attacks. When the attacks ceased, the spatial abilities returned to normal within a few days. Furthermore, in a recent study on mice [31], the seizures were triggered by pentylenetetrazole (PTZ). The changes in cognitive abilities and synaptic plasticity were observed with the Morris water maze and electrophysiological methods (e.g., LTP). Later, a hippocampal dissection was performed. The study revealed that the attacks impaired learning and memory abilities. LTP was also decreased and the expression of inflammatory cytokines in the hippocampus (interleukin-1β (IL-1β) and IL-6, as well as tumour necrosis factor alpha (TNF-α)) were increased. The changes in synaptic plasticity were caused primarily by IL-1β. The decrease in LTP in the hippocampus was also demonstrated by a study in adult rats [32], who were also given PTZ to trigger status epilepticus. Excitatory postsynaptic potentials around the CA1 region in the hippocampus were measured. The decrease in LTP was interpreted as a consequence of the impairment of molecular mechanisms of neuronal plasticity, including mechanisms associated with N-Methyl-D-Aspartate (NMDA) receptors or changes in their subunits.

Taken together, the underlying mechanisms of cognitive impairment may be due to ion channel failure, inflammation factors or factors influencing neuroplasticity. It is possible that more than one factor is involved in individual patients. However, more studies are needed to better address this question.

### 4.2. Epileptiform Activity and Cognitive Impairment

Epileptiform activity is presented as characteristic graphoelements shown on the electroencephalography (EEG) (sharp waves, spikes, spike-and-wave complexes). Simplified, it can be classified into ictal (during a seizure), postictal (after a seizure) and interictal (between two seizures) activity. Epileptiform activity is often associated with memory impairment, mental retardation, communication and behavioural disorders, and impaired attention [34]. 

Furthermore, epileptiform activities can contribute to transient or chronic deficits depending on a number of factors. These are, for example, multiple recurrences, intensity, the age of the person, the type of therapy used to prevent seizures and its effectiveness. The long-term effects of interictal epileptic discharges, which accumulate over time, can lead to significant changes in cognitive functions, especially in the ability to learn and in memory [9]. Children and adolescents whose brains are still developing are particularly exposed. In addition, frequent seizures also lead to a decrease in the power and the frequency of theta waves [23,30]. Theta waves are neural oscillations of frequency from 4 to 8 Hz. Theta rhythm can be recorded in the hippocampus and surrounding limbic structures during the REM phase of sleep. It is closely related to cognitive processes [35].

Three studies [36,37,38] based on intracranial EEG have shown how epileptic activity can alter the normal course of cognitive processes with the disturbance of the large-scale brain network. Initially, short-term memory functions were tested in a study in rats [36] whose hippocampal spikes on an intracranial EEG were triggered by pilocarpine infusion. Epileptic activity had the greatest effect on the processes involved in memory, which were slowed down. The mere presence of spikes on intracranial EEG had no effect on the maintenance of information in memory. Later, Kleen et al. [37] performed a similar study on people with epileptiform activity in the hippocampus area, in which they came to the same conclusions as in animal experiments. Furthermore, Ung et al. [38], using intracranial EEG and memory tests, found that cortical structures in the left temporal lobe were particularly sensitive to the occurrence of interictal epileptic activity during memory coding. In certain areas specific to this memory process, the adverse effects of a single spike could be evaluated. They also found that spikes formed outside the epileptic seizure area had a greater effect on memory than spikes in the seizure area. The results confirmed that the pathological areas of the cortex are already fundamentally dysfunctional and that the interictal activity of the resulting spikes interferes with the cognitive processes associated with a particular part of the brain tissue. The results of these studies suggest that suppressing interictal epileptic activity in the brain could potentially improve memory function and other cognitive abilities.

### 4.3. Cognitive Impairment in Epilepsy and Social Connection

Epilepsy is a disease with an unpredictable course, which can mean certain limitations in social activities for people with this disease. In addition, PWE are still facing stigmas in society [39]. 

Due to cognitive impairment, many PWE have difficulty communicating and establishing interpersonal relationships, for which social cognition is an important factor. Social cognition is the ability to understand the social environment, its organization and relationships and the ability to recognize the intentions and emotions of other people [40]. The impairment of memory, executive functions and attention disorders can prevent people from communicating fluently. The capacity and the speed of information processing are reduced, which can hinder their social inclusion and adaptation. Therefore, transient and chronic cognitive impairment in PWE is a risk factor for poorer social inclusion [41].

Two studies investigated the ability of PWE to recognize emotions. In a study of 74 people with temporal lobe epilepsy, Amlerova et al. [42] used several tests to assess the recognition of emotions on people’s faces and to assess social cognition. The results were also compared to patients after the surgical treatment of temporal lobe epilepsy. The study revealed that PWE performed significantly worse than healthy controls, both in recognizing emotions and in interpreting social interactions. The surgical treatment did not affect the deterioration or improvement of social cognition. The limitations of this study are a relatively small sample of subjects; the fact that they used only one relatively simple test for assessment of social cognition (The Awareness of Social Inference Test—TASIT) (therefore, their conclusions cannot be generalized to the whole social cognitive domain); and the fact that they did not consider the effect of AED treatment. Another observational study [43] was conducted on 43 subjects with focal epilepsy compared to the healthy group. Subjects were shown videos that accurately mimicked real complex social interactions among people. The results showed that PWE had no difficulty in recognizing positive emotions (joy); on the contrary, they were less likely to recognize negative emotions (anger, fear and disgust). The limitations of this study are that they used patients with migraine as a control group, where social cognition has not yet been well studied; that the education did not completely match between subjects and the control group; and that they did not consider AEDs’ effect on cognition. Both studies provided additional evidence that PWE have difficulties with certain aspects of social cognition. They especially have difficulties with correct identification of negative emotional states. It was also suggested that onset of epilepsy, duration of the disease and early brain injury or structural changes in the brain influenced social cognition. However, another explanation of the effect of early epilepsy onset may be that some children with epilepsy can be socially stigmatized early on, which limits social interactions and, consequently, limits social learning.

Furthermore, in a study by the USA Centre for Disease Prevention and Control (CDC) [44], which included 2207 PWE, a number of psychosocial problems were identified. The study revealed significantly higher unemployment rates, lower incomes, lower levels of education, a higher probability of a single life and a poorer lifestyle (obesity, inactivity and smoking) in PWE than in other subjects without epilepsy. In another study, Rai et al. [45] found that PWE had an increased incidence of social phobia and agoraphobia (fear of open spaces), generalized anxiety disorder, depression and a higher rate of suicidal thoughts than the general population without epilepsy or people with other diseases such as asthma and diabetes. However, both of these studies were cross-sectional, meaning they did not allow for direct associations among variables and they did not consider possible effects of mood disorder in the analysis. Additionally, they did not classify reported cases of epilepsy by seizure type, severity, or aetiology.

Despite the limitations, the results of the studies included in this review clearly demonstrate that PWE may need some social support. One of the possibilities are national chapters of the ILAE [46] and national patients’ organisation. 

### 4.4. Effect of Antiepileptic Drugs on Cognitive Impairment

Numerous studies [12,47,48,49,50] show that cognitive impairment is more common in PWE who take more AEDs. As with all pharmacological agents, patients taking AEDs also show more pronounced side effects, when combining more than one AED, or when they are taking higher doses of the drugs [51]. In a study by Martin et al. [47], who tested elderly subjects with epilepsy, those receiving polytherapy performed worse on all tests than subjects receiving monotherapy or healthy subjects. Similar findings were shown in another study a year later [48] based on the same subjects, where polytherapy with AED was also considered as a risk factor for cognitive impairment. Piazzini, on the other hand, considered the effect of AED polytherapy a consequence of pharmacokinetic and pharmacodynamic changes that appear with age [49].

One of the larger studies included 834 PWE [52]. More than a half of the subjects received two or three different AEDs. The majority received lamotrigine or levetiracetam. The study revealed that the likelihood of cognitive impairment increases with each additional AED. Cognitive impairment was more evident in the area of executive functions. Based on the results, the recommendation was given that, whenever possible, no more than two AEDs should be combined in the treatment. 

Furthermore, studies show [53,54] that older generations of AEDs (e.g., carbamazepine, valproate and phenytoin) cause significantly more side effects and negatively affect cognitive functions compared to newer groups, with the exception of topiramate. Lamotrigine [55,56] and levetiracetam [57,58] proved to be better options with less impact on cognitive processes. Topiramate had the most adverse effects on cognition [59]. 

Additionally, two separate studies [55,56] compared the effects of lamotrigine, topiramate and carbamazepine on cognitive functions. In both studies, lamotrigine proved to be less harmful, especially in the area of verbal fluency and attention. 

In a smaller study, Gomer et al. [57] compared cognitive impairment in PWE on topiramate or levetiracetam therapy. Levetiracetam treatment showed no deterioration in any of the tested cognitive areas (attention, verbal and visual–spatial long-term, short-term and working memory, and executive functions). On the other hand, people receiving topiramate achieved below-average results in all areas. In the group of people treated with levetiracetam, even improved attention was recognized. Moreover, Piazzini et al. [58] came to similar conclusions. During levetiracetam therapy, improved attention and verbal fluidity was found in a sample of 38 PWE. However, the study did not take drug doses or the disease itself into consideration, which could have affected the results.

Witt et al. [59] compared the cognitive functions in 880 PWE who received topiramate, any other type of AED, or did not receive therapy. All PWE were compared to a healthy group. The results showed that poorer verbal fluidity was associated with more concomitant AEDs, lower education and topiramate treatment. In addition, verbal fluidity was negatively affected by high drug doses and long-term treatment.

In contrast to the aforementioned studies, Taylor et al. [60] showed that cognitive functions in PWE were worse than in healthy controls even before starting treatment with AEDs. Although there are not many similar studies, this shows there is a wide variety of risk factors (e.g., seizure frequency, duration of epilepsy and interictal abnormalities) which can affect cognition even before diagnosis in PWE. In addition, most of the studies that investigated AEDs’ cognitive side effects did not consider all those other factors caused by epilepsy itself.

In evaluating the results of the aforementioned studies, we also have to consider certain methodological limitations. Loring [61] cites three main factors. The first factor is the inability to randomly divide the treatment regimen, as people with a more resistant form of epilepsy are more likely to try newer drugs and the impact on their cognitive abilities is not representative of the general population of PWE. On the other hand, successful treatment reduces the intensity and frequency of seizures and at least partially eliminates the negative effects of the seizures on cognitive abilities [62]. Secondly, another factor is the difficulty in determining comparable doses of drugs because the doses are individually adjusted. Different doses may have different effects on the cognitive function. The third factor is the impracticability of double-blind studies with AEDs [61]. 

### 4.5. Effect of Epilepsy on Cognitive Abilities

Four studies [47,48,49,50] investigated cognitive impairment in older people with focal epilepsy. Martin et al. [47] included 25 PWE and 27 healthy volunteers over 60 years of age. Individuals with a history of alcohol or illicit substance abuse, people with psychiatric illness or a life-threatening condition in the past year (cancer, bone injury, or heart disease) and people with a history of progressive encephalopathy or any other progressive brain disease were excluded. All PWE had been diagnosed with focal impaired awareness seizures (with or without history of secondary generalized seizures). In both groups, the cognitive abilities in the areas of memory, concentration and attention, executive functions and language were assessed. PWE performed worse on all tests than healthy individuals. Cognitive impairment was statistically significantly associated with the duration of epileptic seizures and the age at the onset of epilepsy. 

Moreover, in a study [48] based on a sample of subjects from the above-mentioned study [47], the cognitive abilities of older PWE were compared to a group of elderly people with mild cognitive impairment. Again, PWE performed worse in the areas of attention, executive functions and language, but not in the area of memory. The poor results of executive functions stood out in particular. A limitation of this study was a small sample of PWE. Furthermore, similar results were obtained in a recent study involving predominantly people with temporal lobe epilepsy [50]. PWE performed worse on testing verbal and visual memory, executive functions, language and attention. In addition to polytherapy with AED, which we mention in the previous subchapter, anxiety was also considered as a risk factor for cognitive impairment in some areas (especially language and visual spatial abilities). In contrast to the aforementioned research studies, Piazzini et al. [49] did not consider the influence of education, the duration of epilepsy, the age at the onset of epilepsy, the frequency of seizures and the aetiology on cognitive processes. They also found no statistically significant differences between the groups in working memory testing. 

Some other studies have focused on a specific type of epilepsy and its impact on cognitive functions. Adults of different ages were included. For instance, Black et al. [63] compared people with temporal lobe epilepsy to people with psychogenic non-epileptic seizures (PNESs). People with a history of mild head injury, depression, or substance abuse were not excluded from the study. The number of attacks was estimated based on the medical history. The main limitation of the research study was the lack of a control group. Working memory and executive functions were tested. The sample included 207 people with temporal lobe epilepsy and 216 people with PNESs. They found that an early onset of seizures significantly affected the working memory and executive functions in both groups. People with the early-onset temporal lobe epilepsy achieved the worst results. In another study [64], difficulties in executive functions and attention were more prominent in the PNES group than in the epilepsy group. The epilepsy group had significantly lower results in memory tasks. However, people with PNESs are associated with unreliable test results [65]. A possible explanation to why people with PNESs were found to have similar cognitive impairment to PWE is that many people with PNESs do not put in maximal effort during neuropsychological assessments due to motivation difficulties. They also often suffer from personality disorders. These patients were found to have some structural abnormalities in the brain. Therefore, it would be worth including them in future research on cognitive impairment [65]. In contrast, in many studies [12,47,48,49,50,60,63,66], the results of PWE on cognition stayed relatively consistent.

Furthermore, another study investigated how frontal lobe epilepsy affects executive functions [66]. In PWE, executive functions are frequently impaired. These include divided attention, which allows a person to adapt quickly and effectively to different situations. The study included 30 people with frontal lobe epilepsy and 30 healthy volunteers. All PWE had clear evidence of the onset of an epileptic seizure in the frontal lobe, clinical features characteristic of seizures in the frontal lobe and normally developed intelligence. Testing was performed by showing faces of different emotions (angry or happy) and different ages (young or old). They found that people with frontal lobe epilepsy had weaker switching abilities than healthy individuals and were less able to categorize the emotions displayed. The results may indicate impaired social cognition. The results are consistent with the fact that the frontal lobe plays an important role in the functioning of executive functions, social skills and emotion regulation. The limitations of the study were the small sample of subjects and only two different forms of emotion shown at the task of categorizing emotions. We did not find any similar research study to compare the results. 

In a recent study, Wang et al. [12] examined various factors that could affect cognitive impairment in PWE of different types. Education level, age, frequency of seizures, type and number of AEDs and depression were considered. The sample included 257 adults diagnosed with epilepsy. The results of PWE were compared with a reference range of neuropsychological tests validated for mild cognitive decline in Alzheimer’s dementia. The results showed that the overall cognitive function in PWE, when using the Montreal Cognitive Assessment (MoCA) and Clinical memory scale, was lower than a certain reference range and this indicates that the tests were sensitive even to mild memory impairment. In other tests that were used in the study (Mini-mental state examination, Digital symbol test, Verbal fluency test, Digit span test), PWE fell into the normal range. Such results were attributed to the low sensitivity of these tests for mild cognitive impairment in PWE. PWE had problems with verbal fluency, attention, executive functions and psychomotor abilities. A high level of education, good disease control, treatment with a single AED and a healthy mental state have been shown to be protective factors of cognitive functions. 

The study by Taylor et al. [60] included people who did not yet receive treatment for epileptic seizures and people on failed AED monotherapy. Everyone with acute symptomatic seizures, a history of progressive neurological disease and structural changes visible on CT or MRI of the brain were excluded from the study. Poorer results were achieved on 13 out of 16 neuropsychological tests. Thus, it can be concluded that people with newly diagnosed epilepsy can already have significant cognitive impairments in the areas of delayed or direct memory recall, executive functions and psychomotor speed compared to healthy individuals. Cognitive impairment, especially in the area of memory, can occur in PWE without structural abnormalities on MRI, with only a few recorded epileptic seizures and before starting regular antiepileptic therapy. 

The limitation of most of the presented studies is that some have not taken into account a wide variety of factors that could influence cognitive impairment. Due to small sample sizes, the results vary among the studies. 

## 5. Conclusions

Cognitive impairment in PWE is a consequence of several factors, such as the early onset of epilepsy, frequent seizures, frequent interictal discharges, low level of education and polytherapy. Recurrent seizures have a strong effect, primarily on the hippocampus, and can cause deficits in brain plasticity. 

Nevertheless, almost all confirm the negative impact of polytherapy on cognition in PWE. These are mainly AEDs of older generations and topiramate. However, it is crucial to take the prescribed therapy consistently to achieve the best seizure control possible. Given that, in many studies, levetiracetam was found to be a drug that had improved the area of attention in patients, it would also make sense to investigate whether some AEDs can even slow the development of cognitive decline in specific areas and in what concentrations. 

The main limitation of most current clinical research is the small sample of subjects. Ideally, all PWE would have a neuropsychological assessment to accurately determine the extent and nature of cognitive impairment. In the future, studies on cognitive impairment in PWE should include larger and well-defined samples. 

We may conclude that PWE may have impaired memory, executive functions, attention and social skills in comparison to healthy subjects. Beside the best medical treatment, PWE may also benefit from the national patients’ organisation and the ILAE. 

## Figures and Tables

**Table 1:** An overview of the research works included in the chapter.

Author (by Year)	Sample Size (*n*)	Main Findings	Limitations
Martin et al. (2005)	*n*(PWE) = 25 *n*(control) = 27	Older PWE performed worse in all cognitive areas. Those who received a higher number of AEDs performed worse, especially in the area of attention and memory recall.	Small sample. Included mostly PWE with the disease lasting an average of >30 years. Limited access to diagnostic information about PWE.
Griffith et al. (2006)	*n*(PWE) = 26 *n*(control) = 26	Compared with individuals diagnosed with MCI, older PWE performed worse on cognitive ability tests. The higher number of AEDs was associated with poorer results, especially in the area of executive functions.	Small and heterogeneous sample. The sample mostly consisted of a preliminary study of the quality of life of elderly adults with epilepsy and did not represent the general population of elderly PWE. The sample of people with MCI was taken only from the tertiary level of health care.
Piazzini et al. (2006)	*n*(PWE) = 40 *n*(control) = 40	Older people with focal epilepsy performed worse on cognitive ability tests than controls. Increased number of AEDs was associated with cognitive impairment.	Small sample. Only people with focal epilepsy were included in the sample.
Black et al. (2010)	*n*(PWE) = 207 *n*(control) = 216	The earlier the onset of epilepsy, the greater the impact on cognitive functions (working memory and executive functions), regardless of the spreading of the seizures. Cognitive impairment was affected by the frequency of the seizures.	Only temporal lobe epilepsy and psychogenic epilepsy were compared. Comorbidities such as past injuries, depression and drug abuse were not considered. The duration of epilepsy was not completely determined and was only an estimate of patients’ reporting.
Taylor et al. (2010)	*n*(PWE) = 155 *n*(control) = 87	PWE performed worse, especially in the areas of memory, psychomotor speed and executive functions. Cognitive ability was not associated with the number of seizures, type of epilepsy, or mood. In newly diagnosed PWE, cognitive impairment was evident before the treatment was initiated.	More appropriate control group needed. Larger sample required. The psychological response and adjustment to a new diagnosis of epilepsy that could affect test results was not assessed.
Gul et al. (2015)	*n*(PWE) = 30 *n*(control) = 30	Poorer ability to switch between tasks in PWE of frontal lobe—impaired executive function. Impaired ability to categorize emotions on the face in PWE—impaired social cognition. PWE of frontal lobe had a poorer inhibitory mechanism to control interference during tasks.	A larger sample is needed. The task of categorizing emotions should consist of a wider range of emotions.
Ozer et al. (2015)	*n*(PWE) = 20 *n*(PNES) = 11*n*(control) = 20	Verbal learning and memory scores, long-term memory and total recognition test scores were significantly lower in PWE than in the controls. In repeat cognitive tests, significant progress was found in verbal fluency of the PNES group. No significant differences were determined in the epilepsy group.	A larger sample is needed. Focused more on PNES than PWE. Study was analysed using non-parametric tests.
Miller et al. (2016)	*n*(PWE) = 38 *n*(control) = 29	PWE performed worse in almost all cognitive areas. A higher number of AEDs was associated with poorer language and visual spatial abilities. Anxiety in PWE was associated with poorer results in visual memory.	Requires a more diverse and a larger sample. Only people over 55 years were included.
Wang et al. (2020)	*n*(PWE) = 257 * compared with the normal reference ranges	People with generalized epileptic seizures performed worse on tests than those with other types of epilepsy. Level of education, frequency of seizures, type of AED and depression affected cognitive functions in PWE. Those who were taking a single AED performed better on the tests.	There was no control group.

PWE—people with epilepsy; AED—antiepileptic drug; MCI—mild cognitive impairment, PNES—psychogenic non-epileptic seizures.

## Data Availability

The data that support the findings of this study are available from the corresponding author, M.R., upon reasonable request.
